# Sex-Based Disparities in Treatment and Healthcare Utilization in Patients with Ulcerative Colitis: A Systematic Review and Meta-Analysis

**DOI:** 10.3390/jcm13247534

**Published:** 2024-12-11

**Authors:** Yash R. Shah, Sneha Annie Sebastian, Dushyant Singh Dahiya, Manesh Kumar Gangwani, Jinendra Satiya, Adishwar Rao, Ramy Mansour, Hassam Ali, Omar Al Ta’ani, Sumant Inamdar, Meer A. Ali, Mohammad Alomari

**Affiliations:** 1 Department of Internal Medicine, Trinity Health Oakland, Wayne State University, Pontiac, MI 48341, USA; 2 Department of Internal Medicine, Azeezia Medical College, Kollam 691537, India; 3 Division of Gastroenterology, Hepatology & Motility, The University of Kansas School of Medicine, Kansas City, KS 66103, USA; 4 Department of Gastroenterology and Hepatology, University of Arkansas for Medical Sciences, Little Rock, AR 72205, USA; 5 Department of Internal Medicine, Yuma Regional Medical Center, AZ 85364, USA; 6 Department of Internal Medicine, Guthrie Medical Center, Sayre, PA 18840, USA; 7 Department of Gastroenterology and Hepatology, Trinity Health Oakland, Pontiac, MI 48341, USA; 8 Division of Gastroenterology, Hepatology & Nutrition, Brody School of Medicine, East Carolina University, Greenville, NC 27834, USA; 9 Department of Internal Medicine, Allegheny Health Network, Pittsburgh, PA 15212, USA

**Keywords:** ulcerative colitis, sex-based disparities, drug therapy, surgery, healthcare utilization

## Abstract

**Background**: Sex-related treatment disparities are well-documented across various medical conditions, yet their impact on the management of inflammatory bowel disease (IBD) remains underexplored. This study aims to investigate sex-based differences in the management of ulcerative colitis (UC), focusing on both medical and surgical approaches and examining whether biological sex correlates with variations in healthcare utilization. **Methods**: A systematic search was conducted across multiple databases, including MEDLINE (via PubMed), Google Scholar, the Cochrane Library, and ScienceDirect, to identify studies on sex differences in ulcerative colitis (UC) management up to April 2024. Statistical analysis was performed using RevMan 5.4, with a random-effects model to combine odds ratios (OR) for both primary and secondary outcomes. The study is registered with PROSPERO (CRD42024537750). **Results**: The meta-analysis included eight observational studies involving 47,089 patients (51.9% females). There were no statistically significant sex differences in biologic therapy use (OR 0.89, 95% CI: 0.69 to 1.15, *p* = 0.36) or corticosteroid use (OR 1.17, 95% CI: 0.89 to 1.54, *p* = 0.27). However, females were less likely to use immunomodulators compared to males (OR 0.89, 95% CI: 0.85 to 0.94, *p* < 0.0001). There were no significant differences in surgical interventions, including total abdominal colectomy. Females had higher annual UC-related hospitalizations compared to males (OR 1.41, 95% CI: 1.22 to 1.64, *p* < 0.00001). **Conclusions**: In conclusion, while biologic and surgical treatments showed no significant sex differences, disparities were noted in immunomodulator use and hospitalization rates, underscoring the need for sex-specific UC management strategies.

## 1. Introduction

Ulcerative colitis (UC) is a chronic inflammatory disease of unknown etiology, characterized by recurrent inflammation of the colonic mucosa. The treatment choice depends on the severity, disease phenotype, localization, and the course of the disease, ranging from medical therapy to surgical options [1]. Socio-demographic factors, including education, income, and sex, influence disease outcomes and impact healthcare access and utilization. While extensive research has examined sex- and gender-specific differences (sex/gender-specific medicine; SGM) in treatment strategies and adherence for conditions like cardiovascular disease, obesity, and diabetes, a comparable focus has not been applied to inflammatory bowel disease (IBD) [2,3,4]. Dedicated studies focusing on this aspect are notably scarce, with available data being sporadic and often secondary to studies centered around different primary endpoints. Gender-specific research emerged in the late 1990s, and sex/gender-specific medicine is a growing discipline. Its goal is to enhance patient outcomes by recognizing sex- and gender-based differences in prevention, clinical manifestations, diagnostics, therapeutic approaches, prognosis, psychosocial impacts, and interactions with the healthcare system [5,6].

The prevalence of IBD differs between the sexes, with distinct patterns emerging for each disease subtype. For Crohn’s disease (CD), females are more commonly affected, with some studies reporting a female-to-male ratio as high as 4:1 [7,8,9]. In contrast, UC is more prevalent in males, with consistent evidence indicating that males are more frequently diagnosed with UC compared to females [7,8,9]. According to studies, the incidence of UC is similar between sexes until middle age; however, after age 45, there is a male predominance in disease incidence. The pathogenesis of UC is driven by the complex interactions of four key factors: environmental exposures, genetic predisposition, immune dysregulation, and likely intestinal dysbiosis. Recent research also suggests that intrinsic (and potentially extrinsic) sex hormones may play a significant role in the development of UC [7]. Additionally, changes in UC incidence are particularly pronounced around the age of menopause in females, with some studies suggesting that menopausal hormone replacement therapy may be associated with an increased risk of developing UC [7].

Genetic predisposition to IBD may also exhibit sex-specific biases. Several susceptibility loci for both CD and UC have been identified on the X chromosome, including haplotypes of the toll-like receptor 8 (TLR8) [7]. These findings support the hypothesis that gene–environment interactions play a crucial role in shaping the baseline susceptibility to IBD, influenced by both sex and age. Additionally, hormone-driven epigenetic modifications that impact immune regulation, and which vary with age, may contribute to the observed sex-based differences in disease incidence for both CD and UC. As a result, there is a clear sex difference in the incidence of IBD, which could influence management strategies [7]. Evidence also supports the presence of sex-based differences in treatment approaches for IBD, highlighting the need for tailored care that accounts for these disparities.

There is a pressing need for further research in sex/gender-specific medicine related to IBD, particularly in the areas of drug therapy, surgical management, and healthcare utilization. Our meta-analysis aims to address this gap by examining sex-based disparities in UC treatment, focusing on variations in medical and surgical management as well as healthcare utilization.

## 2. Materials and Methods

We followed the Cochrane Handbook for Systematic Reviews of Interventions protocol and the reporting standards set by the Preferred Reporting Items for Systematic Reviews and Meta-Analysis (PRISMA) 2020 statement [10,11]. Also, the protocol is registered in PROSPERO (CRD42024537750).

We used the PICO framework to examine sex-based treatment disparities in the management of UC in adult patients. Specifically, the study focused on differences in medical therapy and surgical management between males and females with UC, as well as healthcare utilization patterns. The study addressed the following components:

Population: Adult patients (aged 18+) diagnosed with UC;
Intervention: Treatment strategies, including biologics, corticosteroids, immunomodulators, and surgical interventions (total abdominal colectomy);
Comparison: Comparison of treatment outcomes and healthcare utilization between male and female patients;
Outcomes: Sex-related differences in medication use, surgical management, and healthcare utilization, as detailed below.

### 2.1. Hypotheses

Based on existing literature and the objectives of this study, we hypothesize that there are significant sex-based disparities in the management of UC. Specifically, we expect that females with UC will be less likely than males to be prescribed immunomodulators, that there will be no significant sex differences in the likelihood of undergoing surgical interventions, and that females will experience higher rates of UC-related hospitalizations compared to males. These hypotheses will be tested through a systematic review and meta-analysis of relevant studies, employing statistical methods to compare sex-based differences in medical therapy, surgical interventions, and healthcare utilization patterns.

### 2.2. Search Strategy

We carried out a comprehensive search across multiple databases, including MEDLINE (via PubMed), Google Scholar, the Cochrane Library, and ScienceDirect, to identify studies on how sex influences treatment strategies—both medical and surgical—in the management of UC and its effect on healthcare utilization. The search included all available literature up to April 2024. Additionally, we manually examined the references in the selected articles to find any further relevant studies. To enhance the quality of our research, we also contacted the study authors for additional data and insights. Our search strategy utilized a blend of keywords and Medical Subject Headings (MeSH) terms relevant to “inflammatory bowel disease”, “IBD”, “ulcerative colitis”, “UC”, “sex”, “sex difference”, “gender disparities”, “women”, “medical therapy”, “biologics”, “immunomodulators”, “surgery”, “colectomy”, “hospitalization”, “outpatient clinic visit”, and “healthcare utilization”. Detailed search strategy is available in Supplementary Table S1.

### 2.3. Study Selection

**Figure 1:**
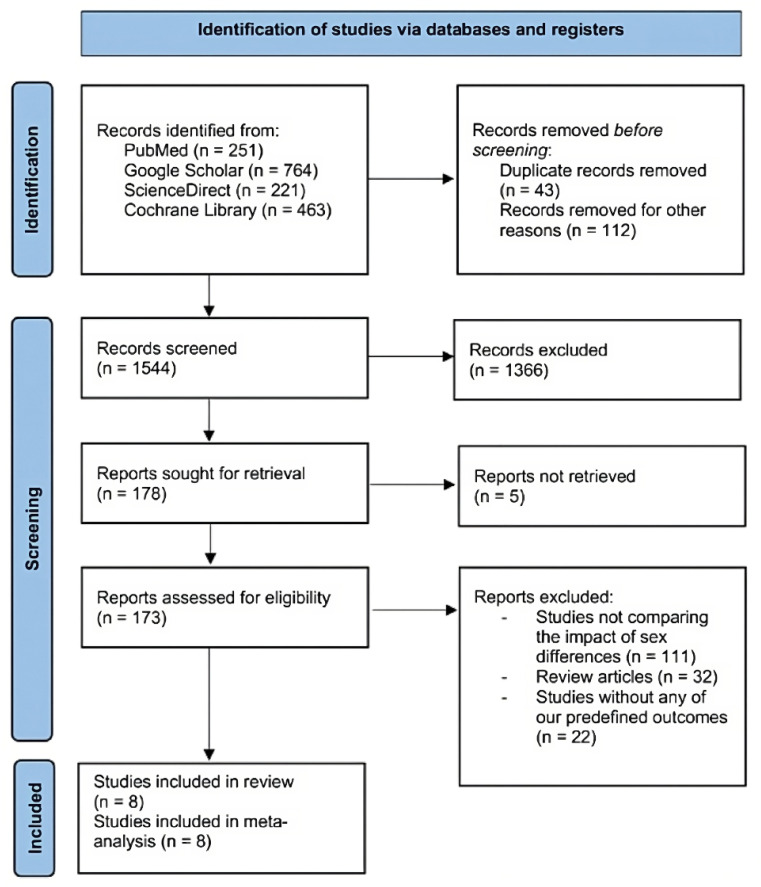
PRISMA flowchart of the study selection process [11].

Following the initial search, which yielded 1699 articles, including 221 articles from ScienceDirect, 251 from PubMed, 764 from Google Scholar, and 463 from the Cochrane Library, we conducted a two-step screening process. After assessing 1544 titles and abstracts against predefined inclusion/exclusion criteria, 178 articles underwent a second stage of full-text screening (Figure 1). Cohen’s Kappa statistic was used to assess the inter-rater reliability for study selection, yielding a Kappa value of 0.9305 [12]. This result indicates near-perfect agreement between raters. The inclusion criteria were centered on both randomized controlled trials and observational studies examining the influence of sex on various treatment strategies and/or healthcare utilization in adult patients aged 18 or older diagnosed with UC, with a minimum follow-up of 6 months or more and published in English. Studies reporting at least one of the predefined outcomes of our study were considered, while exclusions comprised research conducted on animals, case reports, editorials, conference abstracts, reviews, and articles not written in English.

### 2.4. Main Outcomes

Primary outcomes: Examination of sex disparities in medical therapy, including exposure to biologics, corticosteroids use, and immunomodulator use, as well as sex differences in surgical management, specifically assessing the likelihood of undergoing any type of surgery and total abdominal colectomy.

Secondary outcome: Investigation of sex-based disparities in healthcare utilization, particularly focusing on the rate of UC-related hospitalization.

### 2.5. Data Extraction

Upon completing the study selection process, two independent reviewers (YS and SAS) meticulously extracted pertinent information covering various key aspects. These variables included study characteristics such as authors, publication year, country of origin, participant details (particularly age and sample sizes in different groups), follow-up duration, and details regarding study methods and design. Moreover, detailed data on study outcomes were systematically collected.

### 2.6. Risk of Bias

To assess potential biases in the observational studies analyzed, we applied the revised Cochrane Risk of Bias tool for non-randomized studies of exposures (ROBINS-E) [13]. This evaluation was carried out independently by two authors (YS and SAS), who classified the risk of bias as low, high, or uncertain. Any differences in their assessments were resolved through discussion with the senior author (MA). We did not evaluate publication bias because our analysis involved fewer than 10 studies.

### 2.7. Data Synthesis and Statistical Analysis

The meta-analysis of outcomes was conducted using Cochrane Review Manager (RevMan, version 5.4) [14]. Statistical analyses involved calculating the odds ratio (OR) for dichotomous outcomes using the random-effects model. The overall effect was assessed using the Z-value, 95% confidence interval (CI), and probability value (*p*-value). A random-effects model was used to account for both within-study and between-study variability, providing a more conservative and generalized estimation of the overall effect across the included studies. Heterogeneity among studies was assessed using Higgins’s statistical model with I^2^ values [10]. Interpretation of I^2^ values was as follows: <25% indicated low heterogeneity, 25–50% moderate heterogeneity, and >50% high heterogeneity [10]. All *p*-values were determined using a significance threshold of 0.05, indicating statistical significance at the 95% confidence interval (CI).

## 3. Results

### 3.1. Included Studies and Patient Characteristics

The final analysis included eight observational cohort studies comprising 47,089 patients, of whom 24,446 were female and 22,643 were male [15,16,17,18,19,20,21,22]. The average age of the patients included in the analysis was 35.7, with a follow-up duration ranging from 1 to 20 years. Patient data were obtained from a multinational cohort across North American and European nations, including the USA, Canada, Germany, The Netherlands, and Portugal. In five out of the eight included studies, sex-related differences were the primary focus. When examining basic demographic characteristics, some studies noted a higher prevalence of family history of IBD in the female cohort compared to the male cohort. Additionally, a few studies found that male UC patients were more frequently former smokers than female UC patients. However, some studies did not identify any significant sex difference in smoking status. The basic characteristics of each study are concisely summarized in Table 1.

### 3.2. Quality Assessment

**Figure 2:**
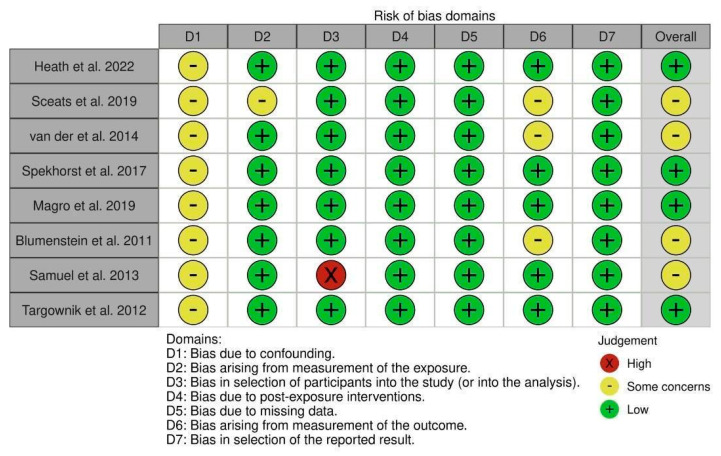

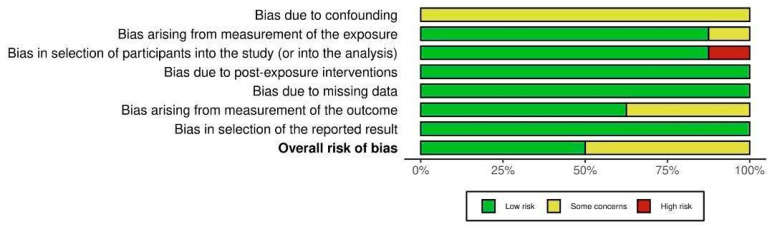
Risk of bias assessment using ROBINS−E tool [15,16,17,18,19,20,21,22].

The included studies primarily exhibited low risk of bias or had some concerns, as depicted in Figure 2. Bias arising from confounding was a common concern in all included observational studies. Notably, in the study by Samuel et al., the selection of participants posed a substantial risk of bias due to an unexpectedly high proportion of individuals employed in healthcare roles, introducing a potential source of bias [21]. Additionally, a few studies raised concerns about bias arising from the measurement of exposure and outcome.

### 3.3. Analysis of Outcomes

**Figure 3:**
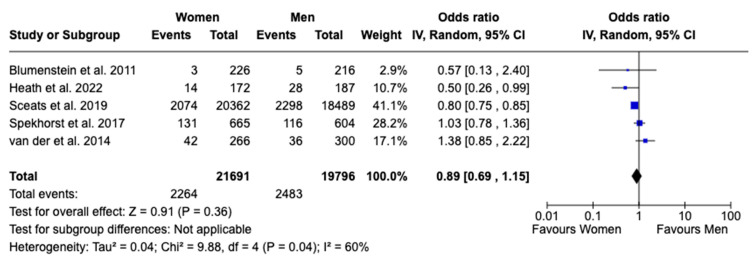
Forest plot for exposure to biologics [15,16,17,18,20].

**Figure 4:**
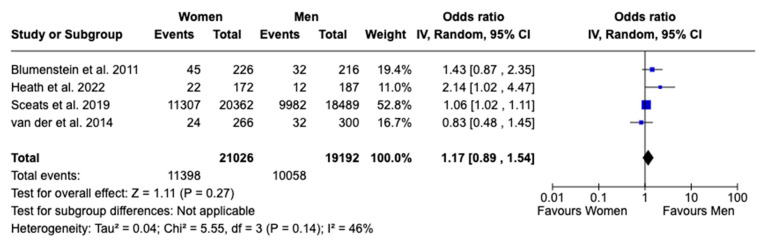
Forest plot for corticosteroids use [15,16,17,20].

In the comparison of biologics use between females and males with UC, five studies were included [15,16,17,18,20]. The analysis found no significant difference between the sexes, with an OR of 0.89 (95% CI: 0.69 to 1.15, *p* = 0.36, I^2^ = 60%), suggesting that sex does not play a major role in the use of biologics (Figure 3). Similarly, combining findings from four studies [15,16,17,20], the use of corticosteroids did not show a statistically significant difference, with an OR of 1.17 (95% CI: 0.89 to 1.54, *p* = 0.27, I^2^ = 46%), indicating comparable usage rates between females and males, although moderate heterogeneity was observed (Figure 4).

**Figure 5:**
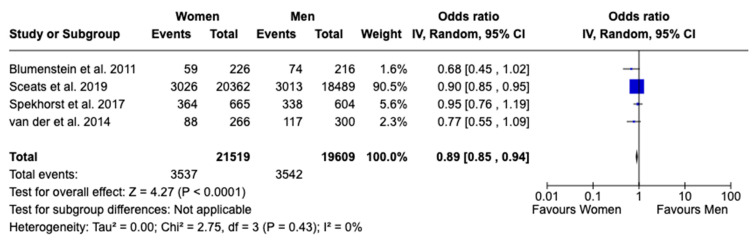
Forest plot for immunomodulator use [16,17,18,20].

However, a statistically significant difference was observed in the use of immunomodulators between females and males with UC, with females being less likely to use these medications. The analysis, which included four studies, showed an OR of 0.89 (95% CI: 0.85 to 0.94, *p* < 0.0001, I^2^ = 0%), indicating no significant heterogeneity across studies (Figure 5). This suggests that the observed difference in immunomodulator use between females and males is consistent across the included studies, reinforcing the reliability of the finding. In effect, this corresponds to a 11% lower likelihood of immunomodulator use in females compared to males, underscoring a significant sex-based disparity in treatment.

**Figure 6:**
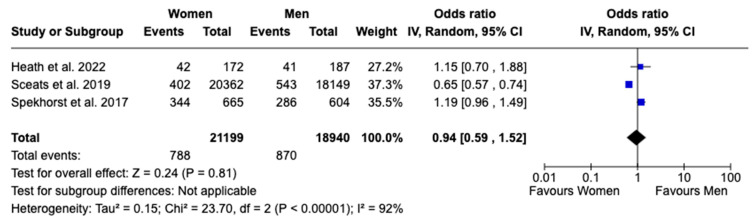
Forest plot for any type of surgical intervention [15,16,18].

**Figure 7:**
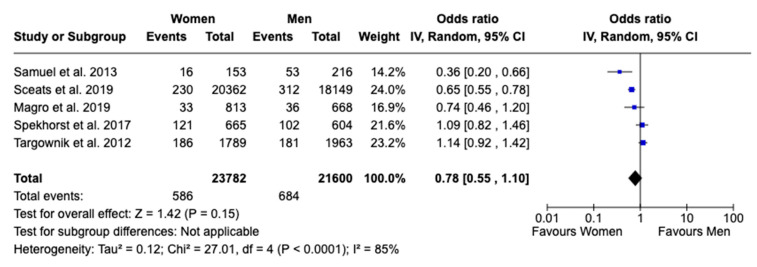
Forest plot for total abdominal colectomy [16,18,19,21,22].

**Figure 8:**
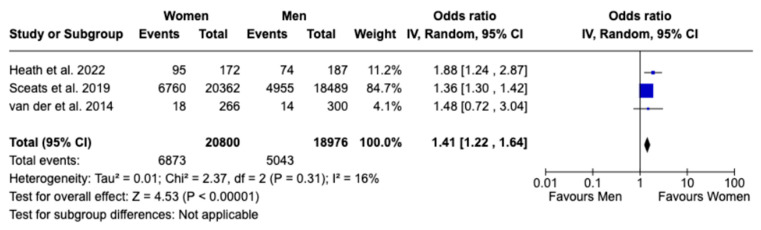
Forest plot for rate of hospitalization [15,16,17].

Regarding surgical interventions, no statistically significant sex difference was found in patients receiving any type of surgical treatment, with an OR of 0.94 (95% CI: 0.59 to 1.52, *p* = 0.81; I^2^ = 92%), nor in patients undergoing total abdominal colectomy, with an OR of 0.78 (95% CI: 0.55 to 1.10, *p* = 0.15; I^2^ = 85%), despite high heterogeneity observed in both analyses (Figure 6 and Figure 7). In analyzing the disparities in healthcare utilization, the annual UC-related hospitalization showed a statistically significant difference between females and males, with females experiencing 41% higher rates of hospitalization, as evidenced by an OR of 1.41 (95% CI: 1.22 to 1.64, *p* < 0.00001; I^2^ = 16%). This finding indicates a notable sex-based disparity with low heterogeneity, suggesting that the result is consistent across the studies included in the analysis (Figure 8). Given the limited number of studies, a publication bias assessment was not conducted.

### 3.4. Sensitivity Analysis

We performed a sensitivity analysis to identify potential sources of heterogeneity. The leave-one-out analysis revealed notable variations in the pooled OR when individual studies were excluded. This indicates that some studies significantly influenced the overall effect estimate. For the use of biologics, excluding two specific studies [17,18] resulted in eliminating heterogeneity completely, with an OR of 0.80 (95% CI: 0.75 to 0.85, *p* < 0.00001; I^2^ = 0%) (Supplementary Figure S1) [17,18]. Similarly, for corticosteroids, a leave-one-out analysis of a single study showed a significant reduction in heterogeneity, leading to no observed heterogeneity, with an OR of 1.07 (95% CI: 0.97 to 1.17, *p* = 0.17; I^2^ = 5%) (Supplementary Figure S2) [15]. These results underscore the significant role of these studies in contributing to the overall heterogeneity observed.

## 4. Discussion

To the best of our knowledge, this is the first meta-analytic study comparing the sex-based differences in UC management and healthcare utilization among adult patients over 18 years of age. Our findings reveal a statistically significant disparity in the use of immunomodulators, with females being less likely to use these medications compared to males. Additionally, females experienced a higher annual rate of UC-related hospitalizations, highlighting notable sex-based disparities in treatment and healthcare utilization. However, no significant differences were found between sexes concerning surgical interventions or the use of biologics and corticosteroids. The only meta-analysis on this topic by Rasmussen et al. reviewed 67 studies involving patients with IBD, including both children and adults [23]. In their analysis, 77.6% of the studies included patients with CD, and 71.6% included patients with UC, though studies focusing specifically on adult UC patients were limited [23]. In contrast, our study concentrated exclusively on adult patients diagnosed with UC. Rasmussen et al. found that many studies on IBD did not report statistically significant sex differences in the likelihood of undergoing surgery or hospitalization [23]. Regarding the use of biologics, the study found that female sex was a strong predictor of non-adherence, while males were more likely to receive systemic treatments, including biologics, compared to females. They emphasized the importance of considering sociodemographic factors, particularly sex differences, in future research and clinical practice for IBD patients [23].

Research shows that males are more likely to undergo surgery for UC compared to females; however, there is no significant difference between sexes in the type of operation performed, as the choice between laparoscopic and open surgery does not vary significantly by sex [24]. Samuel et al. identified male gender and the decade of diagnosis as independent predictors of colectomy [21]. Another study highlighted male gender as a significant risk factor for both early and late colectomy [22]. On the other hand, females were more likely to receive corticosteroid treatments, whereas males were more likely to be treated with biologic medications [20]. Lesuis et al. reported that the use of biologic therapy for UC was more common in males than in females, with rates of 2.5% for males and 1.4% for females [25]. Similarly, another study found that males with UC were more frequently treated with immunosuppressive medications compared to females (34.3% vs. 26.3%) [20]. In contrast, our analysis of data from four studies shows that females with UC are significantly more likely to use immunomodulators than males. Additionally, females received anti-tumor necrosis factor treatment less frequently than males, with rates of 1.4% compared to 2.5% [20]. This suggests that males were more often treated with approaches aimed at long-term disease management or surgical cure. In contrast, females tended to receive treatments focused on managing symptoms reactively, such as corticosteroids and narcotic pain medications, rather than proactive disease management [16]. The literature suggests several reasons for this finding in males: (a) a greater risk of severe disease; (b) lower adherence to corticosteroids and aminosalicylates; and (c) a tendency to avoid prescribing immunosuppressants to females of childbearing age [20]. Overall, studies suggest that males are more likely to receive proactive treatment for UC management, while females are more often treated reactively to manage symptoms [16].

Age is an important confounding factor when examining sex differences. Studies suggest that age influences the prescription of certain medications, particularly biologics, with females over 55 being less frequently prescribed these treatments [15]. Additionally, Zelinkova et al. highlighted the significance of reproductive plans in managing IBD in their analysis of age-related trends [26]. In a cohort of 61 IBD patients, including 21 women with UC, about one-third had to adjust their medication due to active reproductive plans [26]. Another important aspect to consider is that Hepatosplenic T-cell lymphoma (HSTCL), though rare, is a potential risk associated with the use of tumor necrosis factor inhibitors (TNFis) in treating IBD. Identified risk factors for HSTCL in IBD patients undergoing anti-TNF therapy include male gender and being under the age of 35 [27,28]. This underscores the significant role that age plays in the risk profile.

An individual participant data meta-analysis by Agrawal et al. examining gender-based differences in response to TNFi therapies for UC found that male patients had a significantly lower likelihood of achieving clinical remission and mucosal healing compared to women [29]. The evaluation of clinical remission is subjective because it relies on patient-reported symptoms and has a limited correlation with mucosal healing, which is a more objective indicator of inflammation. The observation that males have lower odds of achieving clinical remission compared to females in both treatment and placebo groups may indicate sex differences in the perception and reporting of symptoms [29]. Supporting this, a retrospective study by Nasuno et al. involving 125 patients with corticosteroid-refractory UC showed that females were more likely to achieve clinical remission at one year with infliximab, reinforcing earlier findings [30]. In contrast, a large cohort study of 471 UC patients from the Rhine-Main region in Germany reported significantly higher remission rates in male patients compared to females, with rates of 69.4% versus 58.0% (*p* = 0.013) [20]. This highlights significant sex-based disparities in UC treatment outcomes. The role of sex as a potential risk factor for increased disease activity and a more complicated disease course in UC has been underexplored. Further research is needed to clarify the impact of sex differences on disease progression and management.

Adherence to therapy is critical for managing chronic diseases and maintaining effective remission. Non-adherence is a widespread issue that affects both sexes [31]. Historical studies have shown lower adherence rates among males with IBD, while recent findings suggest that young females may experience even greater adherence challenges [32,33]. However, there is limited data specifically addressing adherence issues in UC. Regarding postoperative complications, sex appears to have a significant impact on outcomes. A study examining sex-related differences in patients undergoing ileal pouch anal anastomosis (IPAA) found that females were more likely to experience bowel obstruction (20.8% vs. 16.7%, *p* = 0.02) and pouch-related fistulas (10.9% vs. 7.6%, *p* = 0.001) [34]. These results suggest that sex may influence the risk of certain postoperative complications.

Previous research on sex differences in IBD healthcare utilization is limited. The available literature suggests that women with UC are more inclined to seek healthcare services than men. Sceats et al. reported that women were slightly more likely to visit the emergency room for UC (9.1% vs. 8.6%) [16]. Conversely, men had a marginally higher average number of annual UC-related outpatient visits (3.0 vs. 2.7, *p* < 0.001). Hospitalization rates for UC, however, were comparable between men and women (15.4% vs. 15.1%) [16]. Regarding seeking care from specialists, there were some differences: females were slightly more likely to see an internal medicine physician compared to males (88.7% vs. 87.4%), with no significant differences in rates of gastroenterologist consultations [16]. Although females underwent less surgical therapy for UC, they were more likely to consult with a colorectal or general surgeon (27.3% vs. 23.5%) [16].

The observed disparities in the management and healthcare utilization of patients with UC may arise from a complex interplay of biological, socio-cultural, and healthcare system-related factors that vary across regions. For instance, regional disparities in access to specialized IBD care, healthcare infrastructure, and cultural norms regarding gender roles could influence treatment patterns and hospitalization rates. Women were less likely to use immunomodulators, which may reflect differences in healthcare-seeking behavior, physician prescribing preferences, or concerns about side effects, particularly regarding childbearing potential. Additionally, higher hospitalization rates among women may indicate differences in disease perception, symptom reporting, or diagnostic thresholds. In regions where disparities were observed, factors like varying insurance coverage, gender bias in clinical decision-making, and differential patient engagement could contribute to these findings.

One of the strengths of this study is its large sample size, which increases statistical power and strengthens the generalizability of the findings. The inclusion of a diverse patient population further enriches the analysis, offering a deeper understanding of how gender differences manifest across various groups of UC patients. Additionally, the study demonstrates low heterogeneity in certain outcome measures, underscoring the consistency and reliability of the results. This homogeneity in the data increases confidence in the observed sex-specific disparities, making the findings more robust and likely applicable to the broader UC population. Overall, our study highlights important trends in sex-based disparities in the management of UC, providing a strong foundation for future, more rigorous research—such as randomized controlled trials—that can build upon our findings. However, it is important to note that the study has several limitations, which are discussed below.

Future research should prioritize understanding how gender differences impact UC treatment and healthcare utilization. Sex-related disparities may serve as important explanatory variables and potential confounders, influencing treatment outcomes and complication rates. A deeper investigation into both patient- and provider-level factors contributing to the observed treatment variations between women and men is needed. Additionally, exploring potential intervention strategies to address these disparities is crucial. Developing targeted educational programs for both patients and healthcare providers could raise awareness and promote more equitable treatment practices. Future studies should also include diverse populations to explore how intersecting factors such as race, socioeconomic status, and geographic location may influence treatment disparities.

## 5. Limitations

This study has several limitations. A significant limitation of our analysis is the predominance of observational studies, which inherently carry a risk of confounding bias. Observational studies, which analyze data collected without controlled experimental conditions, are vulnerable to unaccounted variables that can skew the relationships between factors. This limitation may affect both the accuracy and generalizability of our findings. Additionally, we observed moderate to significant heterogeneity in some of the outcomes, which could further affect the generalizability of the results. The variability in study designs, populations, and measurement methods contributes to this heterogeneity and should be considered when interpreting the findings. Furthermore, the studies included in our analysis often used the terms “sex” and “gender” interchangeably without providing clear definitions, which could introduce additional ambiguity and affect the consistency of the results. In this review, we used the term “sex” to describe biological sex.

## 6. Conclusions

Our study highlights significant sex-based disparities in the treatment and healthcare utilization of patients with UC. While no notable sex differences were observed in the use of biologic therapies or surgical interventions, we identified disparities in the use of immunomodulators and hospitalization rates. These findings underscore the influence of sex-specific factors in UC treatment patterns and healthcare utilization. Further research is warranted to better understand these disparities and their implications for clinical outcomes.

## Figures and Tables

**Table 1:** Baseline characteristics of the included studies.

Author	Country and Year	Study Design	Sample Size (Female/Male)	Mean Age (Years)	Follow-Up	Outcomes
Heath et al. [15]	Canada 2022	Cohort (Single-center)	359 (172/187)	-	7 years	- Exposure to biologics in men versus women - Corticosteroid use men versus women - Surgical intervention (any type) men versus women - Rate of hospitalization in men versus women
Sceats et al. [16]	USA 2019	Retrospective cohort	38,511 (20,362/18,149)	Women: 43 Men: 42.1	3.30 years (range 1–8 years)	- Primary outcome: Sex difference in medical (Corticosteroids use, biologics, immunomodulators, opioids, 5-ASA) and surgical therapy (total abdominal colectomy, total proctocolectomy with ileostomy, total proctocolectomy with ileal pouch) - Secondary outcome: Healthcare utilization
van der et al. (COIN study) [17]	Netherlands 2014	Cohort (Multicenter)	566 (266/300)	Women: 46 Men: 54	2 years	- Gender difference in healthcare utilization and costs - Sex difference in medical therapy
Spekhorst et al. (DUTCH IBD Biobank Study) [18]	Netherlands 2017	Prospective cohort (Multicenter)	(665/604)	Women: 42 Men: 49	1–4 years	- Sex difference in surgery rates
Magro et al. [19]	Portugal 2019	Cohort (Multicenter)	1481 (813/668)	40	12 years	- Sex difference in colectomy
Blumenstein et al. [20]	Germany 2011	Prospective cohort (Multicenter)	442 (226/216)	45.5	2 years	- Gender difference in medical therapy
Samuel et al. [21]	USA 2013	Cohort	369 (153/216)	35.7	13.9 years	- Cumulative incidence of hospitalization - Cumulative incidence of colectomy
Targownik et al. (UMIBDED study) [22]	Canada 2012	Retrospective cohort	3752 (1789/1963)	37	20 years	- Actuarial rates of colectomy at 1, 5, 10, and 20 years

## Data Availability

This study is a meta-analysis of already published studies and the analysis of the data is included in the main manuscript.

## Supplementary Materials

The following supporting information can be downloaded at: https://www.mdpi.com/article/10.3390/jcm13247534/s1. Table S1: PubMed Search Strategy. Figure S1: Sensitivity Analysis for Biologics Use. Figure S2: Sensitivity Analysis for Corticosteroids Use.
