# Supplementary material for: Sex-Based Disparities in Treatment and Healthcare Utilization in Patients with Ulcerative Colitis: A Systematic Review and Meta-Analysis
Source: J Clin Med. 2024 Dec 11;13(24):7534. doi: 10.3390/jcm13247534 (PMC11728175; doi:10.3390/jcm13247534)
Supplement: Supplementary file 1 [file jcm-13-07534-s001.zip › jcm-3331774-supplementary.pdf]

Supplementary Table S1. PubMed Search Strategy.

#1	(“inflammatory bowel disease” [Title/Abstract] OR "IBD” [Title/Abstract]) OR ("ulcerative colitis" [Title/Abstract] OR "UC" [Title/Abstract])
#2	“gender disparities” [Title/Abstract] OR “ulcerative colitis” [Title/Abstract]
#3	(“ulcerative colitis” [Title/Abstract] OR “gender” [Title/Abstract] OR “sex” [Title/Abstract])
#4	((“ulcerative colitis” [Title/Abstract] AND “gender disparities” [Title/Abstract] OR “UC” [MeSH] AND “gender” [MeSH]))
#5	("women” [MeSH] OR "men" [MeSH]) OR ("sex" [MeSH] OR "gender]) OR ("female" [MeSH] OR "male” [MeSH])
#6	("medical therapy" [MeSH] OR "corticosteroids" [MeSH]) OR ("biologics" [Title/Abstract] OR "immunomodulators" [Title/Abstract] OR "tumor necrosis factor inhibitors" [Title/Abstract]) OR (“drug therapy” [Title/Abstract] OR “medical treatment” [Title/Abstract])
#7	#4 AND #5 AND #6
#8	("surgical therapy" [MeSH] OR "surgery" [MeSH]) OR ("colectomy" [MeSH] OR "total abdominal colectomy" [Title/Abstract]) OR ("procedures" [Title/Abstract] OR "complications” [MeSH])
#9	("hospitalisation" [MeSH] OR "emergency visits" [MeSH]) OR ("outpatient visits" [MeSH] OR "costs" [Title/Abstract] OR “healthcare utilisation” [Title/Abstract])
#10	#7 AND #8 AND #9
#11	("adherence" [MeSH] OR "remission" [MeSH]) OR ("treatment adherence” OR “relapse"[MeSH])

Study or Subgroup	Women Events	Women Total	Men Events	Men Total	Weight	Odds ratio IV, Random, 95% CI
✓ Blumenstein et al. 2011	3	226	5	216	0.2%	0.57 [0.13 , 2.40]
✓ Heath et al. 2022	14	172	28	187	0.9%	0.50 [0.26 , 0.99]
✓ Sceats et al. 2019	2074	20362	2298	18489	99.0%	0.80 [0.75 , 0.85]
× Spekhorst et al. 2017	131	665	116	604	0.0%	1.03 [0.78 , 1.36]
× van der et al. 2014	42	266	36	300	0.0%	1.38 [0.85 , 2.22]
**Total (95% CI)**		**20760**		**18892**	**100.0%**	**0.80 [0.75 , 0.85]**
Total events:	2091		2331			

Heterogeneity: Tau² = 0.00; Chi² = 1.98, df = 2 (P = 0.37); I² = 0%
Test for overall effect: Z = 7.16 (P < 0.00001)
Test for subgroup differences: Not applicable

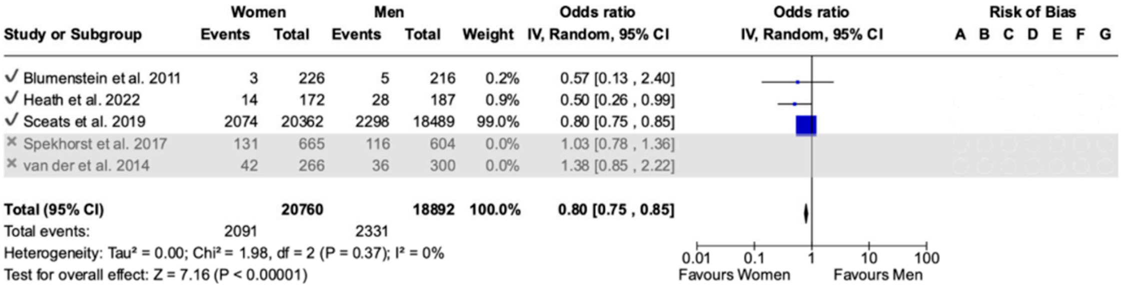

Risk of Bias
A B C D E F G

Risk of bias legend
(A) Random sequence generation (selection bias)
(B) Allocation concealment (selection bias)
(C) Blinding of participants and personnel (performance bias)
(D) Blinding of outcome assessment (detection bias)
(E) Incomplete outcome data (attrition bias)
(F) Selective reporting (reporting bias)
(G) Other bias

Supplementary Figure S1. Sensitivity analysis for biologics use.

Study or Subgroup	Women Events	Women Total	Men Events	Men Total	Weight	Odds ratio IV, Random, 95% CI
✓ Blumenstein et al. 2011	45	226	32	216	3.4%	1.43 [0.87 , 2.35]
× Heath et al. 2022	22	172	12	187	0.0%	2.14 [1.02 , 4.47]
✓ Sceats et al. 2019	11307	20362	9982	18489	93.8%	1.06 [1.02 , 1.11]
✓ van der et al. 2014	24	266	32	300	2.8%	0.83 [0.48 , 1.45]
**Total (95% CI)**		**20854**		**19005**	**100.0%**	**1.07 [0.97 , 1.17]**
Total events:	11376		10046			

Heterogeneity: Tau² = 0.00; Chi² = 2.11, df = 2 (P = 0.35); I² = 5%
Test for overall effect: Z = 1.37 (P = 0.17)
Test for subgroup differences: Not applicable

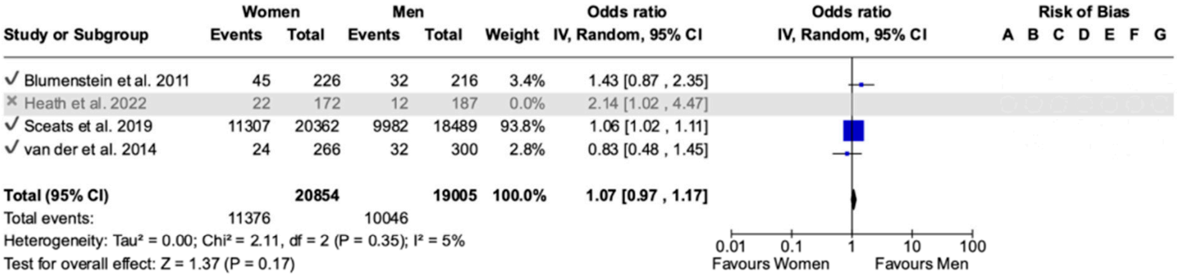

Risk of Bias
A B C D E F G

Risk of bias legend
(A) Random sequence generation (selection bias)
(B) Allocation concealment (selection bias)
(C) Blinding of participants and personnel (performance bias)
(D) Blinding of outcome assessment (detection bias)
(E) Incomplete outcome data (attrition bias)
(F) Selective reporting (reporting bias)
(G) Other bias

Supplementary Figure S2. Sensitivity analysis for corticosteroids use.
